# Self-guided mindfulness and cognitive behavioural practices reduce anxiety in autistic adults: A pilot 8-month waitlist-controlled trial of widely available online tools

**DOI:** 10.1177/1362361320909184

**Published:** 2020-04-08

**Authors:** Sebastian B Gaigg, Paul E Flaxman, Gracie McLaven, Ritika Shah, Dermot M Bowler, Brenda Meyer, Amanda Roestorf, Corinna Haenschel, Jacqui Rodgers, Mikle South

**Affiliations:** 1 City, University of London, London, UK; 2 King’s College London, UK; 3 MAPS Psychology, India; 4 University of Westminster, UK; 5 Newcastle University, UK; 6 Brigham Young University, USA

**Keywords:** anxiety, autism, cognitive-behavioural therapy, online, mindfulness

## Abstract

Anxiety in autism is an important treatment target because of its consequences for quality of life and well-being. Growing evidence suggests that cognitive behaviour therapies and mindfulness-based therapies can ameliorate anxiety in autism but cost-effective delivery remains a challenge. This pilot randomised controlled trial examined whether online cognitive behaviour therapy and mindfulness-based therapy self-help programmes could help reduce anxiety in 54 autistic adults who were randomly allocated to either an online cognitive behaviour therapy (n = 16) or mindfulness-based therapy (n = 19) programme or a waitlist control group (n = 19). Primary outcome measures of anxiety, secondary outcome measures of broader well-being and potential process of change variables were collected at baseline, after programme completion, and then 3 and 6 months post-completion. Baseline data confirmed that intolerance of uncertainty and emotional acceptance accounted for up to 61% of self-reported anxiety across all participants. The 23 participants who were retained in the active conditions (14 mindfulness-based therapies, 9 cognitive behaviour therapies) showed significant decreases in anxiety that were maintained over 3, and to some extent also 6 months. Overall, results suggest that online self-help cognitive behaviour therapy and mindfulness-based therapy tools may provide a cost-effective method for delivering mental health support to those autistic adults who can engage effectively with online support tools.

**Lay abstract:**

Anxiety in autism is an important target for psychological therapies because it is very common and because it significantly impacts upon quality of life and well-being. Growing evidence suggests that cognitive behaviour therapies and mindfulness-based therapies can help autistic individuals learn to manage feelings of anxiety but access to such therapies remains problematic. In the current pilot study, we examined whether existing online cognitive behaviour therapy and mindfulness-based therapy self-help tools can help reduce anxiety in autistic adults. Specifically, 35 autistic adults were asked to try either an existing online cognitive behaviour therapy (n = 16) or mindfulness-based therapy (n = 19) programme while a further 19 autistic adults served as a waitlist comparison group. A first important finding was that 23 of the 35 (66%) participants who tried the online tools completed them, suggesting that such tools are, in principle, acceptable to many autistic adults. In addition, adults in the cognitive behaviour therapy and mindfulness-based therapy conditions reported significant decreases in anxiety over 3 and to some extent also 6 months that were less apparent in the waitlist group of participants. On broader measures of mental health and well-being, the benefits of the online tools were less apparent. Overall, the results suggest that online self-help cognitive behaviour therapy and mindfulness-based therapy tools should be explored further as a means of providing cost-effective mental health support to at least those autistic individuals who can engage effectively with such online tools.

## Introduction

The majority of autistic children, adolescents and adults have one or more associated mental health conditions (Buck et al., 2014; Simonoff et al., 2008), with co-occurring anxiety disorders among the most common concerns. Although prevalence estimates vary across studies, the current consensus is that 40%–50% of autistic individuals meet formal criteria for a co-occurring anxiety disorder (Buck et al., 2014; Van Steensel et al., 2011) compared to 10%–15% in the general population (Bandelow & Michaelis, 2015; Kessler et al., 2012; Wittchen et al., 2011). Although evidence suggests that cognitive behaviour therapy (CBT) and mindfulness-based therapy (MBT) can help reduce anxiety in autism (Cachia et al., 2016; Spain et al., 2015), access to appropriate mental health services is currently inadequate for the autism community, particularly for adults (Lake et al., 2014; Turcotte et al., 2016). Given recent evidence that online mental health support tools can help reduce anxiety in the general population (e.g. Krusche et al., 2013; Powell et al., 2013; Saddichha et al., 2014), the current study examined whether such existing tools could also benefit autistic adults.

A considerable body of evidence has accumulated over the past 15 years, which suggests that psychological therapies that are commonly used to treat mental health difficulties in the general population are also effective for autistic individuals. One-to-one and group-based CBT, for example, has been shown to lead to moderate-to-large reductions in anxiety in autistic youths and adults, similar to the effects observed in the general population (for reviews, see Lang et al., 2010; Spain et al., 2015; Ung et al., 2015). Similarly, mindfulness-based approaches, which are effective in managing a wide range of mental health concerns in the general population (Creswell, 2016; Goldberg et al., 2018; Hofmann et al., 2010), also appear to benefit autistic individuals (Kiep et al., 2015; Sizoo & Kuiper, 2017; Spek et al., 2013; see Cachia et al., 2016 for a review). An increasing understanding of the mechanisms underlying anxiety disorders in autism (see Rodgers & Ofield, 2018; South & Rodgers, 2017; Vasa & Mazurek, 2015; for recent reviews) has played an important role in shaping approaches to psychological interventions. For instance, anxiety is consistently linked to *intolerance of uncertainty* (IU) in autism (e.g. Boulter et al., 2014; Maisel et al., 2016), which is characterised by a fear of the unknown and a tendency to avoid uncertain and unpredictable situations (Carleton, 2012; Carleton et al., 2012). Based on evidence suggesting that high levels of IU are associated with poorer treatment responses to standard CBT in autistic youth (Keefer et al., 2017), Rodgers and colleagues (2017, 2018) have therefore developed a parent-mediated programme (CUES©; Coping with Uncertainty in Everyday Situations) that specifically targets IU. Other authors have tailored CBT approaches to target a broad range of emotion-regulation skills due to the considerable evidence that links anxiety in autism to reduced use of adaptive and/or increased use of maladaptive emotion regulation strategies (for reviews, see Cai et al., 2018; Mazefsky et al., 2013; Weiss, 2014; White et al., 2014). Finally, MBTs have been attracting increasing attention as an approach for managing anxiety in autism, partly because they cultivate present moment awareness and non-judgmental attitudes towards difficult thoughts, feelings and bodily sensations (Farb et al., 2012; Guendelman et al., 2017; Nyklíček & Kuijpers, 2008). This may be particularly effective in the context of autism where anxiety is commonly linked to sensory processing differences and elevated levels of *alexithymia* (*ALX*; see Vorst & Bermond, 2001), which is characterised by difficulties in identifying and describing one’s own emotions (Maisel et al., 2016; Milosavljevic et al., 2016; Nicholson et al., 2019).^1^

Despite the evidence that now exists about anxiety in autism, access to appropriate health care services remains inadequate for the autism community, in particular for adults (Howlin & Moss, 2012; Lake et al., 2014; Maddox & Gaus, 2019; Povey et al., 2011; Turcotte et al., 2016). This is a significant concern considering that approximately two-thirds of all people with autism are adults and the vast majority of them report feeling underserved by mental health services (Camm-Crosbie et al., 2018; Rosenblatt, 2008). Moreover, adults may be at increasing risk of developing mental health difficulties as they grow older due to the impact of cumulative traumatic life events and vulnerabilities to unemployment and financial hardship (Griffiths et al., 2019; Taylor & Gotham, 2016). A major barrier to delivering psychological therapies at the scale required is their cost. Although investment in mental health services would return substantial savings for governments over the longer term (e.g. Chisholm et al., 2016; Layard et al., 2007), it is unrealistic to expect that significant investment will be forthcoming in the near future considering that health services in general, and mental health services in particular, remain under-resourced (Farmer & Dyer, 2016; Goin & Long, 2014). There is an urgent need, therefore, to explore alternative strategies for delivering mental health services to the autism community (and the community at large).

Online- or smartphone-based CBT and MBT programmes, including self-guided tools that do not require the support of a therapist, may offer some solution. Such tools have been shown to be effective in reducing anxiety and other mental health difficulties in neurotypical samples, often to a similar degree to what might be expected from face-to-face interventions (for reviews, see Saddichha et al., 2014; Spijkerman et al., 2016). For instance, Krusche et al. (2013) showed that an online self-guided mindfulness-based programme (www.bemindfulonline.co.uk) that follows the structure of typical face-to-face programmes, yields significant reductions in anxiety, depression and stress that are maintained over at least 1 month. Although rigorous randomised controlled trials are still scarce in this literature, all indications are that online tools will play a significant role in future health care services. In fact, the National Health Service (NHS) in the United Kingdom already endorses certain online support tools such as the MBT programme evaluated by Krusche et al. (2013).

Given the state of current evidence, the principal aim of the present study was to carry out a pilot study to examine whether existing online self-guided CBT and MBT tools could benefit autistic adults in reducing levels of anxiety (primary outcome) and broader mental health difficulties (secondary outcomes). Based on the findings of Maisel et al. (2016) that a combination of IU, ALX and emotional acceptance (EA) accounts for over 60% of the anxious symptoms associated with autism in adults, a secondary aim was to establish whether this finding replicates and whether online CBT and MBT tools would influence these possible process of change variables over time.

## Methods

### Participants

Participants were recruited primarily from an existing research participant database at the host laboratory and through advertisement of the study through adult autism support networks in the south east of the United Kingdom. Participants were therefore self-selecting from the community rather than recruited in the context of a clinical service. Of 72 autistic adults who initially contacted the research team for further information about the study, 54 (75%) ultimately agreed to enrol. They were randomly allocated to one of three conditions that will be described in more detail shortly – a mindfulness-based course (MBT; n = 19), a cognitive behaviour therapy programme (CBT; n = 16) and a waiting list (WL) condition. Thirty-nine participants were enrolled from the existing database, which meant that certain information relating to their diagnosis and cognitive ability was already available. This information was used to stratify randomisation to the different conditions so that groups would be reasonably matched on cognitive ability (intelligence quotient (IQ)) and age. Participants who responded to open advertisements of the study were enrolled sequentially to the three groups. Ultimately, three participants in the MBT group did not start the mindfulness course after returning baseline questionnaires and a further two participants did not complete the programme after starting. In the CBT group two participants did not start the programme and five did not complete it, and in the WL group we lost contact with three participants between time-points 1 and 2. Thus, in the active treatment groups, 76% of all participants who started the MBT (88%) or CBT (64%) programme were retained for follow-up, leading to a final sample size for longitudinal analyses of 39 participants (14 MBT, 9 CBT and 16 WL).

The main inclusion criteria for enrolling in the trial were that participants could provide confirmation that they had received a clinical diagnosis of autism spectrum disorder (ASD) through the UK’s NHS in line with the relevant diagnostic criteria that were in place at the time of diagnosis (e.g. *Diagnostic and Statistical Manual of Mental Disorders* (4th ed.; *DSM*-IV) or *DSM*-5; American Psychiatric Association, 2000, 2013). In addition, they needed to confirm that they were currently not receiving any form of psychological therapy for managing mental health difficulties. Core clinical difficulties were characterised through Module 4 of the Autism Diagnostic Observation Schedule (ADOS; Lord et al., 2000), the adult self-report version of the Social Responsiveness Scale (SRS-2-ASR; Constantino & Gruber, 2012) and the Autism-Spectrum Quotient (AQ; Baron-Cohen et al., 2001). Information about broader cognitive functioning was obtained through the third or fourth edition of the Wechsler Adult Intelligence Scale (WAIS-III-UK or WAIS-IV; Wechsler, 1999, 2008). Some participants did not complete all of these assessments because they either dropped out or because it was difficult to arrange face-to-face appointments due to travel distances. Specific data on socioeconomic status were not recorded. Table 1 provides a summary of all available participant characteristics with participants who dropped out listed separately to those who were retained. Non-completers compared to completers had lower Verbal IQ (t = 2.36, df = 42, p = 0.02; Cohen’s d = 0.80) and demonstrated a greater degree of difficulties in the ADOS Communication domain (t = 2.19, df = 39, p = 0.03; Cohen’s d = 0.73). Among completers, there were no significant group differences on any of these measures.

**Table 1.** Participant characteristics as a function of study condition.

	Mindfulness (n = 14)			CBT (n = 9)			Waitlist (n = 16)			Non-completers (n = 15)		
	n	M (SD)	Range	n	M (SD)	Range	n	M (SD)	Range	n	M (SD)	Range
Gender (M:F)	12:2			8:1			12:4			11:4		
Age (years)	14	42.5 (10.3)	28.6–66.3	8	40.3 (12.7)	26.7–58.0	14	45.7 (13.6)	23.9–64.8	13	43.2 (12.7)	23.7–62.2
Verbal IQ	13	110.6 (13.4)	88–138	6	119.0 (11.0)	103–131	13	123.1 (17.6)	81–143	12	104.8 (15.4)	81–134
Non-verbal IQ	13	111.1 (15.7)	89–136	6	109.2 (14.5)	94–128	13	115.5 (13.5)	84–142	12	103.3 (20.4)	59–128
Full-scale IQ	12	111.7 (12.1)	88–128	6	116.7 (12.5)	99–133	11	117.6 (15.5)	81–135	12	103.6 (17.7)	77–132
ADOS-Comm.	12	2.7 (1.5)	0–5	6	2.2 (1.0)	1–4	12	2.1 (1.1)	1–4	11	3.4 (1.6)	1–6
ADOS-RSI	12	6.2 (2.4)	4–11	6	4.7 (0.8)	4–6	12	5.8 (3.0)	2–13	11	6.4 (2.5)	4–11
ADOS-Total	12	8.8 (3.6)	5–16	6	6.8 (1.7)	5–10	12	7.9 (3.8)	3–17	11	9.7 (2.8)	6–14
AQ	14	32.4 (5.7)	24–39	9	34.2 (6.1)	25–43	16	35.8 (8.7)	16–49	11	32.6 (10.5)	18–47
SRS-SCI	14	66.5 (12.8)	45–86	9	68.2 (6.3)	57–79	16	65.0 (12.4)	36–84	9	71.2 (14.2)	51–90
SRS-RRB	14	66.9 (12.5)	47–87	9	71.3 (9.5)	55–83	16	65.1 (12.9)	40–90	9	74.0 (13.0)	58–90
SRS-Total	14	67.1 (12.8)	47–87	9	69.4 (5.8)	61–80	16	65.4 (12.8)	36–87	9	71.8 (13.5)	53–90

SD: standard deviation; CBT: cognitive behaviour therapy; IQ: intelligence quotient; ADOS: Autism Diagnostic Observation Schedule; Comm.: Communication; RSI: Reciprocal Social Interaction; AQ: Autism-Spectrum Quotient; SRS: Social Responsiveness Scale; SCI: Social Communication and Interaction; RRB: Restricted Interests and Repetitive Behavior.

### Outcome measures

To capture a range of anxiety symptoms, four well-established measures were used as primary outcome measures to, respectively, assess generalised anxiety (*The General Anxiety Disorder-7* – GAD-7; Spitzer et al., 2006), social anxiety (*Liebowitz Social Anxiety Scale* – LSAS; Heimberg et al., 1999), trait anxiety (*State-Trait Anxiety Inventory* – STAI-T; Spielberger et al., 1983) and bodily manifestations of anxiety such as feelings of numbness and dizziness (*Beck’s Anxiety Inventory* – BAI; Beck et al., 1988). Secondary outcome measures included the depression sub-scale of the *Hospital Anxiety and Depression Scale* (HADS-D; Zigmond & Snaith, 1983) and the *Clinical Outcomes in Routine Evaluation – Outcome Measure* (CORE-OM; Evans et al., 2000), which provides a broad index of mental health and well-being including risk to self and others and the impact of mental health symptoms on daily living. Finally, ALX, IU and EA were assessed as possible process of change variables. The sum of the *Identify* and *Describe* subscales of *The Bermond-Vorst Alexithymia Questionnaire* (BVAQ-ID; Vorst & Bermond, 2001) served as the measure of ALX since these domains have previously been shown to be particularly relevant to anxiety in autism (see Maisel et al., 2016). The 12-item *Intolerance of Uncertainty Scale* (IUS-12; Carleton et al., 2007) and the non-reactivity to inner experiences sub-scale of the *Five Facet Mindfulness Questionnaire* (FFMQ-NR; Baer et al., 2006) were used to assess IU and EA, respectively. Further details about each of the questionnaires are provided in the Supplemental Material (S1) along with a summary of their internal consistencies, which were generally strong.^2^

All questionnaires were combined into booklets that were sent to participants by post for each of the four data collection points. The questionnaires were printed in the order shown in Table 2, which ensured that participants were first asked to reflect on their mental health over the past 1 or 2 weeks (BAI, LSAS, HADS, CORE-OM and GAD) before answering questions concerning more general trait characteristics (STAI-T, IUS-12, FFMQ-NR and BVAQ-ID). The questionnaires were printed in a standardised format that represented the different Likert-type scales in the form of boxes that participants were required to tick. Each questionnaire began on a new page with the relevant standardised instructions. In addition to the questionnaires, participants in the two active conditions (MBT and CBT) were also sent ‘diary pages’ at T1, which they were asked to use on a weekly basis to record how they engaged with the practices they learned and to note any concerns, thoughts or feedback about the programmes. Since only 15 participants (65%) returned these pages, however, these data were not analysed and will not be discussed further.

**Table 2.** Descriptive statistics of the key outcome measures at baseline as a function of experimental condition.

	MBCT (n = 14)	CBT (n = 9)	WL (n = 16)	Non-completers (N = 15)
	M (SD)	M (SD)	M (SD)	M (SD)
*Primary outcomes*				
GAD-7	6.1 (4.7)	11.1 (8.1)	9.63 (6.3)	7.7 (6.0)
LSAS	59.6 (31.6)	67.3 (32.6)	60.0 (31.4)	69.0 (30.7)
STAI-T	48.2 (12.2)	52.4 (15.2)	53.7 (14.8)	53.3 (10.7)
BAI	10.2 (7.3)	20.0 (11.0)	16.5 (11.0)	19.0 (12.5)
*Secondary outcomes*				
HADS-depression	6.1 (4.5)	6.7 (4.0)	8.6 (5.9)	7.4 (4.9)
CORE-OM	45.0 (21.0)	49.6 (25.8)	57.6 (28.7)	53.2 (21.8)
*Process of change variables*				
IU	37.7 (12.1)	42.6 (9.4)	40.9 (9.2)	39.1 (11.6)
BVAQ-ID	23.9 (5.2)	24.2 (6.3)	25.3 (7.2)	23.0 (5.3)
FFMQ-NR	20.6 (4.4)	20.3 (5.2)	20.3 (6.5)	17.8 (5.9)

SD: standard deviation; CBT: cognitive behaviour therapy; WL: waiting list; GAD: General Anxiety Disorder; LSAS: Liebowitz Social Anxiety Scale; STAI-T: State-Trait Anxiety Inventory; BAI: Beck’s Anxiety Inventory; CORE-OM: Clinical Outcomes in Routine Evaluation – Outcome Measure; IU: intolerance of uncertainty; BVAQ-ID: Identify and Describe subscales of The Bermond-Vorst Alexithymia Questionnaire; FFMQ-NR: non-reactivity to inner experiences sub-scale of the Five Facet Mindfulness Questionnaire.

### Online mental health programmes

Participants who were randomised to the MBT group were enrolled on the online *Be Mindful* course (https://www.bemindfulonline.com/), which has been reported to yield similar reductions in perceived stress, anxiety and depression as traditional face-to-face mindfulness interventions in the general population (Krusche et al., 2013). The course comprises a total of 10 exercises that are explained in instructional videos and audio files that participants gain access to as they progress through the course. The overall aim of the exercises is to cultivate present moment awareness and non-judgmental attitudes towards thoughts and feelings as they arise. Participants randomised to the CBT group completed the self-help programme *Serenity* (https://serene.me.uk; https://serene.me.uk/kiosk-0/anxiety_menu.php), which was developed in the context of an NHS service with the aim of making CBT more widely accessible for people experiencing anxiety (Slegg et al., 2009). The programme is based on trans-diagnostic CBT principles and aims to help people understand the nature of their anxiety and how to manage it through exercises that are presented in illustrated slides. Participants in both the MBT and CBT groups were encouraged to work through the programmes with the aim of completing the course in 6–8 weeks. Further details about the *Be Mindful* and *Serenity* programmes are included as Supplemental Material (S2).

### Procedure

**Figure 1.**
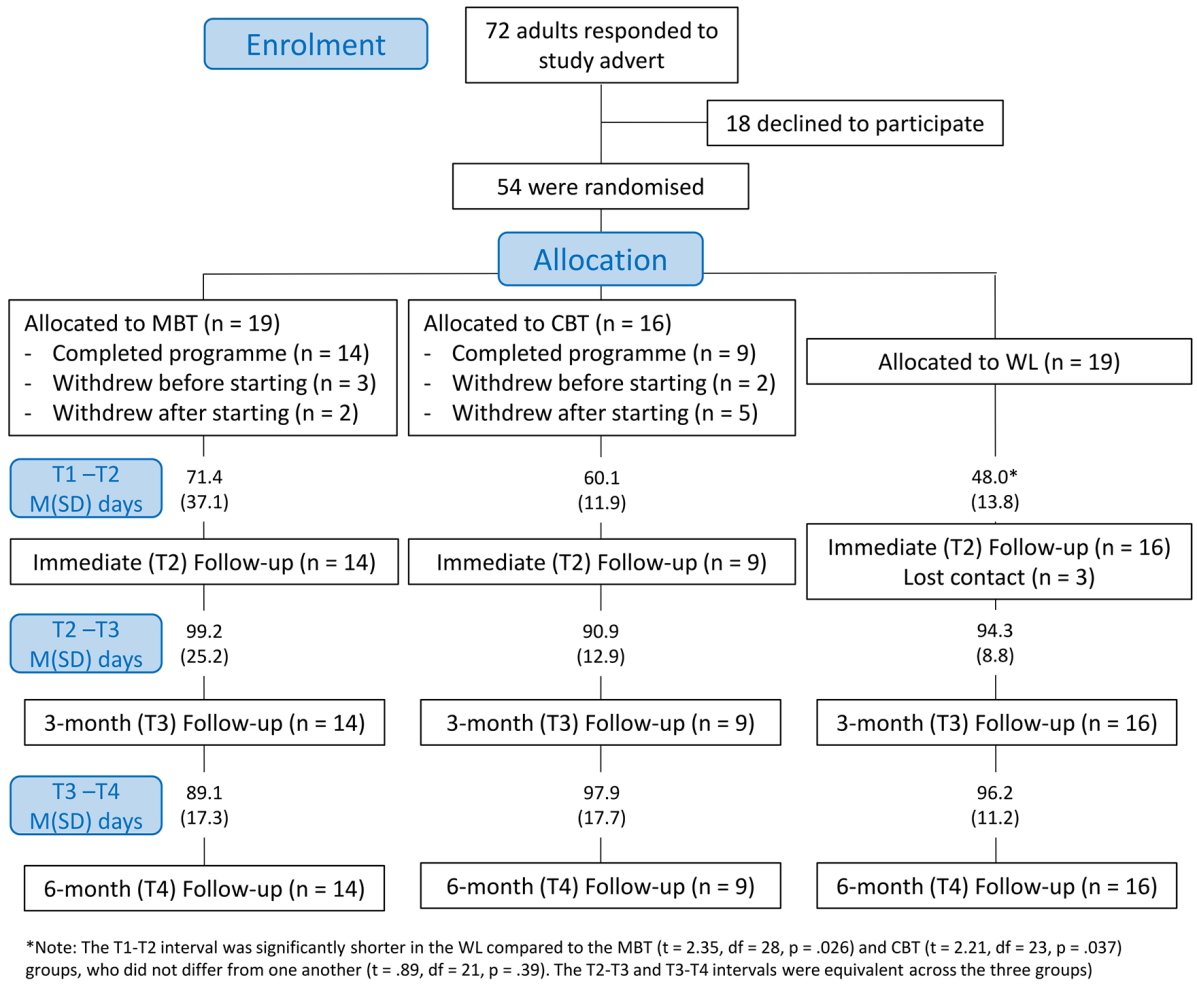
Overview of trial timeline and allocation of participants.

Participants were enrolled in two waves, from June–August 2016 (n = 35) and again from March–April 2017 (n = 19). After providing informed consent, participants were allocated to a group and sent the first questionnaire booklet (T1) by post, along with a pre-paid return envelope for returning the questionnaire booklet to the research team. Once the completed T1 questionnaires were returned, participants in the MBT and CBT groups received instructions on how to access the respective online programmes. Once they confirmed starting the programmes by e-mail or phone, they received weekly phone calls from a member of the research team to monitor and encourage progress, and to answer any questions. Upon course completion, participants were sent the post-intervention (T2) questionnaires, along with copies of the AQ and SRS-2 if scores on these measures were not already available. Efforts were also made at this point to arrange face-to-face appointments to administer the WAIS-IV and ADOS if these were not already on file.^3^ Twelve weeks after participants returned the T2 questionnaires, T3 booklets were sent and a further 12 weeks after these were received the final T4 questionnaires were sent. At the conclusion of the trial period, all participants were offered the opportunity to access the online tools they had not already gained access to. Figure 1 provides an overview of the project timeline including details of the average intervals between the four time-points in the three experimental groups. All study procedures were reviewed and approved by the Psychology research ethics committee of City, University of London in line with the British Psychological Society’s code of ethics and the Declaration of Helsinki.

### Data management and analyses

Missing data were minimised by contacting participants to clarify missing or ambiguous answers to questionnaire items as soon as packs were returned. Of a total of 36,936 questions, only 37 answers could ultimately not be clarified, and these were pro-rated based on the relevant total or sub-factor scores of the remaining items in the questionnaires. However, three participants (two MBT and one CBT) failed to return the T2 questionnaire packs, two participants in the CBT group did not return the T4 questionnaires and for one participant in the WL group the LSAS was not completed at T4 due to an error in preparing the relevant booklet. In order to retain these participants in all analyses, the relatively conservative decision was taken to carry the results of the previous time-point forward to the missing time-point (e.g. carry the results of T1 forward to the missing T2), essentially assuming no change in this period.

In the analyses that follow, we first carefully examine the baseline data to clarify the prevalence of clinically significant levels of anxiety in our sample and to establish the extent to which the suspected process of change variables (IU, ALX, EA) predict baseline levels of anxiety (through correlation and regression analyses). We then turn to our primary aim of examining longitudinal changes in primary and secondary outcome measures through analyses of variance (ANOVAs) and the calculation of indices of *reliable change* (*RC*) and *clinically significant change* (*CSC*) at an individual participant level (Evans et al., 1998; Jacobson & Truax, 1991). RC is demonstrated if reductions in a participant’s anxiety score on a particular questionnaire are greater than the measurement error of that questionnaire,^4^ whereas CSC is demonstrated if this change furthermore moves the participant out of the range of scores that would be considered clinical caseness. In a final ANOVA analysis, we then also examine longitudinal changes in the process of change variables (IU, ALX, EA).

## Results

### Baseline data

Descriptive statistics for all questionnaire measures at baseline are set out in Table 2 as a function of the three experimental conditions, with non-completers listed separately. The data for all questionnaires were normally distributed. Although one-way ANOVAs indicated no significant group differences between completers and non-completers on any of the measures (max t = 1.57; min p = 0.122), or between the three experimental groups (max F = 1.87; min p = 0.164), it is worth noting that the MBT group had considerably lower average GAD-7 and BAI scores at baseline than both the CBT (GAD-7 Cohen’s d = 0.76; BAI Cohen’s d = 1.05) and WL groups (GAD-7 Cohen’s d = 0.64; BAI Cohen’s d = 67). Thus, some differences across the experimental groups in baseline anxiety were apparent.

Table 3 summarises further details about the distribution of scores on the four primary outcome measures of anxiety and the wider secondary clinical outcome measures (HADS-D and CORE-OM) for all groups. All except the STAI-T have well-established clinical cut-off scores to distinguish minimal, mild, moderate and severe levels of symptoms. On the BAI (Beck & Steer, 1990), LSAS (Mennin et al., 2002), HADS (Bjelland et al., 2002) and CORE-OM (CORE Partnership, 2007), the mild symptom range is normally considered a cut-off in clinical practice for further investigation but for the purposes of the current study we consider the moderate and severe levels to indicate clinical caseness because this range of scores is indicative of relevant anxiety disorders with very high probability. For the STAI-T, we specified quartile ranges of scores as indicative of minimal, mild, moderate and severe symptoms with the assumption that scores in the moderate and severe range constitute clinical caseness. In line with the existing literature, the majority of participants in the current sample (72.2%) reported moderate or severe levels of anxiety on at least one of the four anxiety measures and only a small minority (4%) reported minimal symptoms on all measures. Approximately half of the participants met the criterion for clinical caseness on the LSAS, BAI and STAI with 40% meeting this criterion on the GAD. Beyond anxiety, 22% of participants also met criteria for depression on the HADS and 54% reported significant impact of mental health difficulties on well-being and daily functioning on the CORE-OM.

**Table 3.** Summary of the percentage of participants scoring within quartile ranges (minimal, mild, moderate and severe) on the four primary outcome measures of anxiety, and the secondary outcome measures as a function of experimental condition; non-completers are shown separately.

	MBT (n = 14) (%)	CBT (n = 9) (%)	WL (n = 16) (%)	Non-completers (n = 15) (%)	Total (n = 54) (%)
GAD-7					
Minimal (0–4)	50.0	22.2	18.8	33.0	31.5
Mild (5–9)	21.4	22.2	31.3	26.7	25.9
Moderate (10–14)^a^	28.6	11.1	25.0	26.7	24.1
Severe (>14)^a^	0.0	44.4	25.0	13.3	18.5
LSAS					
Minimal (0–30)	28.6	0.0	12.5	0.0	11.1
Mild (31–60)	28.6	55.6	31.3	40.0	37.0
Moderate (61–90)^a^	28.6	11.1	43.8	40.0	33.3
Severe (>90)^a^	14.3	33.3	12.5	20.0	18.5
BAI					
Minimal (0–7)	35.7	11.1	18.8	13.3	20.4
Mild (8–15)	35.7	22.2	25.0	26.7	27.8
Moderate (16–25)^a^	21.4	33.3	37.5	33.3	31.5
Severe (>25)^a^	7.1	33.3	18.8	26.7	20.4
STAI-T					
Minimal (20–35)	21.4	11.1	12.5	13.3	14.8
Mild (36–50)	28.6	33.3	18.8	26.7	25.9
Moderate (51–65)	42.9	22.2	50.0	53.3	44.4
Severe (>65)	7.1	33.3	18.8	6.7	14.8
HADS-D					
Minimal (0–7)	64.3	55.6	50	46.7	53.7
Mild (8–10)	28.6	22.2	12.5	33.3	24.1
Moderate (11–14)^a^	0.0	22.2	18.8	13.3	13.0
Severe (>14)^a^	7.1	0.0	18.8	6.7	9.3
CORE-OM					
Minimal (0–34)	35.7	33.3	18.8	20.0	28.2
Mild (34–50)	21.4	11.1	18.8	26.7	17.9
Moderate (51–84)^a^	42.9	55.6	43.8	46.7	46.2
Severe (>84)^a^	0.0	0.0	18.8	6.7	7.7

MBT: mindfulness-based therapy; CBT: cognitive behaviour therapy; WL: waiting list; GAD: General Anxiety Disorder; LSAS: Liebowitz Social Anxiety Scale; STAI-T: State-Trait Anxiety Inventory; BAI: Beck’s Anxiety Inventory; CORE-OM: Clinical Outcomes in Routine Evaluation – Outcome Measure; HADS-D: depression sub-scale of the Hospital Anxiety and Depression Scale.

a Indicates the threshold we adopt for clinical caseness. On the HADS-D, LSAS, BAI and CORE-OM scores in the mild range are also considered clinically significant but in practice this is typically considered the threshold for further investigation (i.e. clinical caseness is probable). Moderate or severe levels, on the other hand, have very high sensitivity and scores in this range are very likely to indicate clinical caseness.

Table 4 summarises the correlations among the questionnaire measures at baseline, and a number of details are worth highlighting about these data. First, among the anxiety measures, there were strong correlations between the GAD-7, STAI-T and BAI whereas correlations with the LSAS were somewhat less pronounced, especially with the GAD-7. This provides some evidence for convergent validity among the questionnaires that capture non-specific sources of anxiety, while discriminant validity is also demonstrated with respect to the distinction between generalised anxiety and social anxiety. Second, IU was consistently related to all measures of anxiety and to the wider outcome measures of depression and clinical functioning (CORE-OM). The same also applied to the EA measure with the exception that the association with social anxiety (LSAS) was not significant following Bonferroni correction. ALX was less consistently related with the anxiety and secondary outcome measures.

**Table 4.** Bivariate correlations among the questionnaire measures at baseline (T1).

	GAD-7	LSAS	STAI-T	BAI	IU	BVAQ-ID	NR	HADS-D
Primary outcomes (anxiety)								
LSAS	0.335*							
STAI-T	0.761***	0.489***						
BAI	0.752***	0.495***	0.679***					
Process of change variables								
IU	0.570***	0.562***	0.623***	0.563***				
BVAQ-ID	0.245	0.341*	0.403**	0.189	0.324*			
FFMQ-NR	−0.529***	−0.294**	−0.721***	−0.479***	−0.475***	−0.390**		
Secondary Outcomes								
HADS-D	0.563***	0.477***	0.611***	0.511***	0.378**	0.364**	−0.353**	
CORE-OM	0.771***	0.492***	0.868***	0.748***	0.585***	0.325*	−0.658***	0.677***

GAD: General Anxiety Disorder; LSAS: Liebowitz Social Anxiety Scale; STAI-T: State-Trait Anxiety Inventory; BAI: Beck’s Anxiety Inventory; IU: intolerance of uncertainty; BVAQ-ID: Identify and Describe subscales of The Bermond-Vorst Alexithymia Questionnaire; FFMQ-NR: non-reactivity to inner experiences sub-scale of the Five Facet Mindfulness Questionnaire; CORE-OM: Clinical Outcomes in Routine Evaluation – Outcome Measure; HADS-D: depression sub-scale of the Hospital Anxiety and Depression Scale.

* p < 0.05; **p < 0.01; ***p < 0.001 (this level accommodates Bonferroni correction).

Next, a series of regressions were undertaken to establish whether IU and EA are independent predictors of each of the baseline anxiety measures when entered together into regression models. All models (i.e. one for each anxiety measure) were significant (min F = 11.85; all ps < 0.001) and in all except one model IU and EA were independent predictors (β > 0.27; t > 2.17; p < 0.05), explaining a total of 39% of the variance in GAD-7 scores, 35% in BAI scores and 61% in STAI-T scores. The exception was the model with the LSAS as the dependent variable, where only IU was identified as a predictor (β = 0.55; t = 4.15; p < 0.001) with no independent contribution from EA (β = −0.034; t = 0.26; p = 0.796). Full details of these regression analyses are presented as Supplemental Material (S3) and it is worth noting that if the BVAQ-ID measure is added as a predictor alongside IU and EA, it does not add significantly to any of the models.

## Longitudinal data

**Figure 2.**
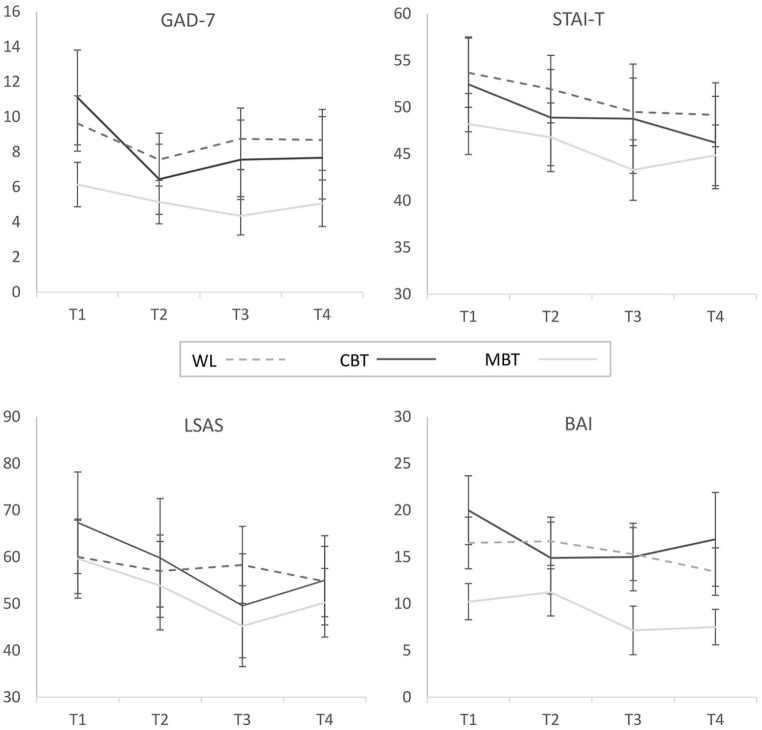
Longitudinal changes in the four primary outcome measures of anxiety over the four time-points as a function of experimental group. Higher scores on all measures reflect a greater degree of anxiety. Error bars represent ±1SE.

Longitudinal changes in the four primary outcome measures of anxiety (GAD-7, LSAS, STAI-T and BAI) over the four time-points are shown in Figure 2. A multivariate analysis of variance (MANOVA) with the four time-points (T1, T2, T3 and T4) as a within-subjects factor and group (MBT, CBT, WL) as a between-subjects factor confirmed a significant main effect of time (F(3,34) = 10.37, p < 0.001; partial η^2^ = 0.48). Although no interaction between time and group was indicated (F(6,70) = 1.63, p = 0.152; partial η^2^ = 0.12), planned comparisons within each group separately showed that the main effect of time across all measures was significant in the MBT (F(3,11) = 8.85, p = 0.003; partial η^2^ = 0.71) and CBT groups (F(3,6) = 7.71, p = 0.018; partial η^2^ = 0.79) with large effect sizes, whereas it was not significant in the WL group (F(3,13) = 1.56, p = 0.248; partial η^2^ = 0.26) where the effect size was small.

**Figure 3.**
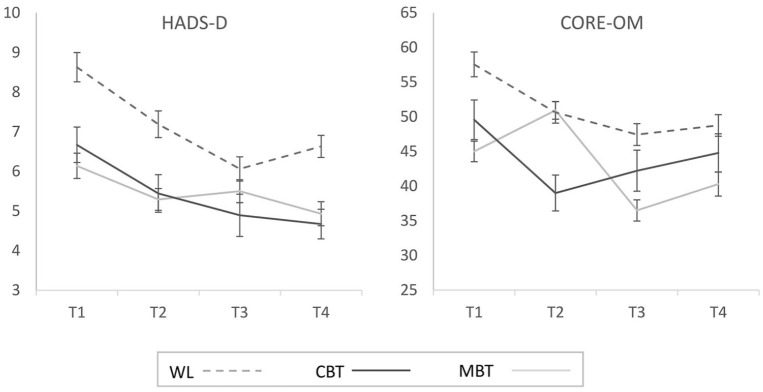
Longitudinal changes in the secondary outcome measures of depression (HADS) and broader clinical functioning (CORE-OM) as a function of experimental group. Higher scores reflect greater levels of depression (HADS) and broader clinical difficulties (CORE-OM). Error bars represent ±1SE.

Figure 3 shows the longitudinal changes in the two secondary outcome measures of depression (HADS-D) and wider clinical functioning (CORE-OM). Repeated measures ANOVAs for each of these measures yielded significant main effects of time (HADS-D: F(3,73.84) = 3.47, p = 0.035, partial η^2^ = 0.09; CORE-OM: F(3,108) = 3.55, p = 0.017, partial η^2^ = 0.09) but no main effect of group or group × time interaction.

To better understand how useful the online resources might be in clinical practice, we next examined the RC and CSC indices for the 28 participants (9 MBT, 7 CBT and 12 WL) who demonstrated clinical caseness on at least one of the anxiety measures or on the CORE-OM at baseline (not enough participants demonstrated clinical caseness on the HADS-D to render this analysis feasible for symptoms of depression). Table 5 summarises these data and shows that at 3-month follow-up over 75% of participants in the MBT (77.8%) and CBT (100%) group demonstrated reliable reductions in at least one of the anxiety measures, with benefits maintained for over 50% of participants at 6-month follow-up. Interestingly, an increasing proportion of the WL group also reported reliable reductions in anxiety over time such that after 6 months there was no clear advantage in the active treatment, compared to the WL group. Further inspection of these data at the level of each individual measure of anxiety (see Supplemental Table S4) showed that reductions in anxiety in the active CBT and MBT groups were most evident for the GAD-7, LSAS and BAI and to a lesser extent the STAI-T. The improvements in the WL group were less consistent across the different measures, with up to only a quarter of participants demonstrating reliable reductions at 3 months on any given measure compared to around 50% in the active groups.

**Table 5.** The percentage of participants demonstrating reliable change (RC) and clinically significant change (CSC) on at least one of the four anxiety measures on which clinical caseness was demonstrated at baseline. Also shown are the percentages of participants who demonstrated RC and CSC on the CORE-OM.

		n	Change in Anxiety			n	CORE-OM		
			T1–T2 (%)	T1–T3 (%)	T1–T4 (%)		T1–T2 (%)	T1–T3 (%)	T1–T4 (%)
RC	MBT	9	33.3	77.8	66.7	6	22.2	33.3	33.3
	CBT	7	71.4	100.0	57.1	5	33.3	33.3	33.3
	WL	12	33.3	41.7	58.3	10	30.8	38.5	23.1
CSC	MBT	9	33.3	66.7	22.2	6	33.3	33.3	16.7
	CBT	7	42.9	57.1	57.1	5	40.0	0.0	20.0
	WL	12	16.7	33.3	25.0	10	30.0	30.0	20.0

CORE-OM: Clinical Outcomes in Routine Evaluation – Outcome Measure; RC: reliable change; MBT: mindfulness-based therapy; CBT: cognitive behaviour therapy; WL: waiting list; CSC: clinically significant change.

Column n indicates the number of participants who demonstrated clinical caseness at baseline.

The CSC data largely paralleled the RC results and showed that over 50% of participants in the MBT (66.7%) and CBT (57.1%) groups demonstrated clinically significant improvements in anxiety at 3-month follow-up, which were maintained for around a third of participants (22.2% in MBT and 57.7% in CBT) until the 6-month follow-up. Again, some improvements were seen also in the WL group but also here such improvements were less consistent across the individual measures (see Supplemental Table 3). Finally, improvements on the CORE-OM were generally less pronounced with up to a third of participants demonstrating significant improvement at 3 and 6 months but there was little indication of advantages in the active MBT and CBT groups compared to the WL group.

**Figure 4.**
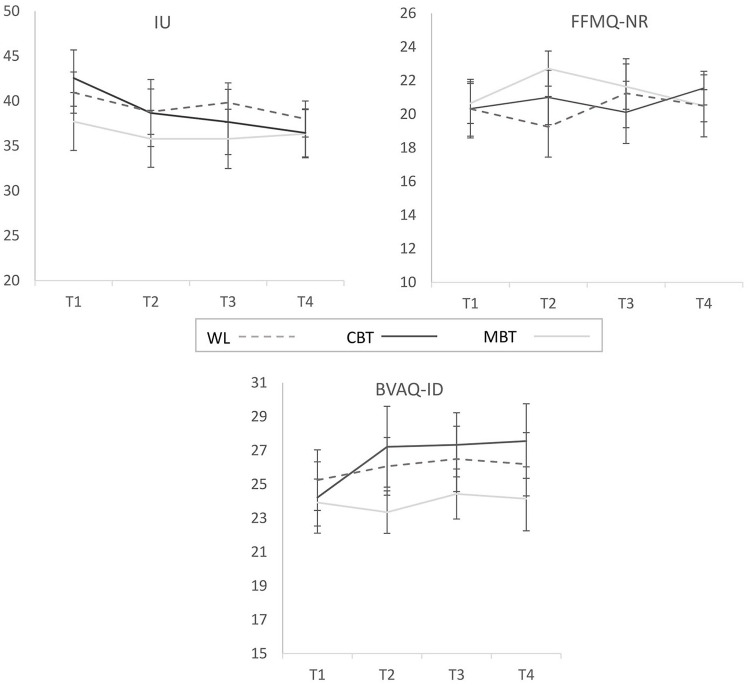
Longitudinal changes in the process of change variables intolerance of uncertainty (IU), non-reactive thinking (FFMQ-NR) and alexithymia (BVAQ-ID) over the four time-points as a function of experimental group. Higher scores on IU and BVAQ-ID reflect greater intolerance of uncertainty and alexithymia, respectively. Higher scores on the FFMQ-NR reflect greater non-reactivity (an adaptive emotion regulation strategy). Error bars represent ±1SE.

Finally, Figure 4 illustrates the changes over time in the three process of change variables. Repeated measures ANOVAs showed that IU decreased significantly over time across the three groups (F(3,108) = 4.50, p = 0.005, partial η^2^ = 0.11), with no significant group × time interaction. There was no significant change in the EA measure (F(3,108) = 0.35, p = 0.79, partial η^2^ = 0.01) and ALX scores unexpectedly increased over time (F(3,108) = 3.01, p = 0.033, partial η^2^ = 0.07), again with no group by time interaction.

## Discussion

To the best of our knowledge, the current study is the first to suggest that widely available online self-help tools that teach people CBT or MBT strategies to manage difficult feelings are generally acceptable to a large number of autistic adults – of 35 adults who were allocated to the online *Be Mindful* (n = 19) and *Serenity* (n = 16) programmes a total of 23 (66%) completed them. Moreover, a significant number of participants who completed the online programmes also demonstrated reliable and clinically significant reductions in anxiety over a 3 month, and to a lesser extent also a 6-month period. Before discussing these findings in detail, we will first briefly consider some implications of the baseline data of the current study.

Maisel et al. (2016) recently showed that the combination of IU, ALX and EA accounts for over 60% of the association between autism and anxiety. The current findings broadly replicate this observation but with an important qualification. In Maisel et al. (2016), ALX, EA and IU were all significant correlates of anxiety in a combined sample of autistic (n = 76) and non-autistic (n = 75) adults. However, when all three factors were considered together, ALX and EA were the most significant predictors of the relationship between anxiety and autism with no independent contribution from IU. By contrast, in the current sample, ALX was only moderately related to anxiety whereas the combination of IU and EA accounted for between 35% and 61% of anxious symptoms across different measures. This discrepancy is most likely a reflection of the fact that the current study included only autistic adults. It is now generally thought that ALX commonly co-occurs with ASD due to shared underlying genetic and neurobiological factors rather than constituting a consequence of (or cause for) core characteristics of autism (see Bird & Cook, 2013; Poquérusse et al., 2018). ALX may therefore be a risk factor for increased anxiety in autism that is expressed through the more proximal causes of IU and EA. This would explain why ALX does not contribute independently to anxiety *within* a group of autistic individuals when IU and EA are taken into consideration, whereas in combined groups of participants it explains a considerable amount of the *between-group differences* in anxiety (as in the study by Maisel et al., 2016). Several studies in the neurotypical literature support this conclusion (see Palser et al., 2018; Pandey et al., 2011). Palser et al. suggested that ALX contributes to anxiety by rendering internal bodily sensations confusing, which implies uncertainty about internal states.

Another important observation in our baseline data relates to the issue of measuring anxiety accurately in autistic adults. Studies of children have shown that overlap between the core clinical characteristics of autism and the symptoms of anxiety can render standardised clinical tools invalid (Kerns et al., 2015; Wood & Gadow, 2010), which may furthermore not be sensitive to autism-specific expressions of anxiety (Kerns et al., 2014). Our baseline data demonstrate good internal consistencies (Cronbach’s alphas >0.90) for all four primary outcome measures of anxiety (see Supplemental Material S1). Moreover, the inter-correlations between the BAI, STAI and GAD-7 (r > 0.67) provides evidence of convergent validity among measures of non-specific sources of anxiety while the lower correlation between LSAS and particularly the GAD-7 (r = 0.34) provides evidence of discriminant validity for measures of generalised versus social anxiety. The fact that both EA and IU were independent predictors of BAI, STAI and GAD-7 whereas only IU predicted LSAS scores lends further support to this point. Thus, instruments that are currently widely used in clinical settings to screen for anxiety disorders in the general adult population can probably be considered valid also for autistic adults, with the caveat that autism-specific presentations of anxiety may be missed (see Kerns et al., 2014, 2017; Rodgers et al., 2016). Importantly, these conclusions need to be further explored in clinical settings and with more representative samples of autistic adults.

The longitudinal data suggest that currently available online self-help tools can help a substantial number of autistic adults learn MBT or CBT strategies to manage clinically significant levels of anxiety. At a group level, participants pursuing the online MBT and CBT programmes demonstrated significant reductions in the primary outcome measures of anxiety with large effect sizes, whereas a WL group demonstrated only minimal improvements. At the level of individual participants, results furthermore showed that over 75% of participants who demonstrated moderate to severe levels of anxiety at baseline reported reliably reduced symptoms 3 months after completing the self-guided CBT or MBT course, and for over 50% these benefits were maintained over 6 months. These findings are in line with studies of face-to-face CBT and MBT interventions (Cachia et al., 2016; Spain et al., 2015) and suggest that it is feasible to deliver such interventions cost-effectively online. Somewhat surprisingly, an increasing number of participants in the WL group also demonstrated reductions in anxiety such that by the final time-point there was no advantage for the MBT and CBT versus the WL group. Observing improvements in WL control groups in intervention studies is not uncommon and may represent ‘spontaneous’ improvement over extended evaluation periods, the utilisation of other sources of support, or growing positive anticipation of gaining access to a potentially effective treatment (e.g. Allexandre et al., 2016; Barkham & Shapiro, 1990; Flaxman & Bond, 2010). Regardless of the source of this observation, the absence of clear group differences at the final time-point was as much a reflection of spontaneous improvements in the WL group as it was due to a fading of the initial benefits for at least some participants in the CBT and MBT groups from 3 to 6 months post-intervention. It will therefore be important for future studies to consider how treatment benefits can best be maintained over prolonged periods, for example, through booster sessions.

In relation to the secondary outcome measures of depression (HADS-D) and broader clinical functioning (CORE-OM), these also demonstrated improvements across time at a group level although here all three groups demonstrated similar gains. This finding is somewhat difficult to interpret because rates of clinically significant levels of depression were relatively low in our sample and because the WL group demonstrated the greatest baseline levels of depression and broader clinical difficulties. Given evidence of wide-ranging mental health benefits from online programmes such as *Be Mindful* (Krusche et al., 2013) and the significant correlations between the primary and secondary outcome measures in the current study (see Table 4), it seems reasonable to expect that future studies would detect clearer benefits in such broader outcomes.

Another important finding in the current study is that 76% of participants who started the CBT or MBT programmes completed them, which suggests that online mental health support tools are generally acceptable to at least those autistic adults who can effectively engage with them. Useful to note in this context is that participant retention was somewhat better for the MBT (88%) than the CBT group (64%), which probably reflects the fact that the *Be Mindful* platform scaffolds continued engagement through weekly e-mail reminders while the *Serenity* programme is entirely self-guided. Although we sought to ensure retention and treatment fidelity through regular phone-contact with participants, this may not promote engagement with online tools as much as more direct scaffolding directly from relevant platforms.

In relation to the possible process of change variables we examined, the results showed that IU significantly decreased across the entire sample over the four time-points whereas ALX surprisingly increased with no change in EA. Closer inspection of Figure 4 suggests that the decreases in IU and increases in ALX were primarily evident in the CBT group, which may indicate that CBT strategies are more effective at targeting these processes of change than MBT. In relation to IU, this would be in line with recent demonstrations that IU can be targeted with CBT strategies (Rodgers et al., 2017) but with respect to ALX one would predict changes to be evident primarily in the context of MBT (Guendelman et al., 2017). More importantly, we would expect to see a reduction rather than an increase in ALX over time (see Norman et al., 2019). The unexpected increase raises an interesting possibility. High levels of ALX may make it difficult for autistic individuals to introspect on the difficulties they have in labelling and understanding inner experiences as emotions with the ensuing uncertainty leading to high levels of anxiety. In learning how to reflect on own emotions and restructure how to think and feel about triggers of anxiety, autistic individuals may become more aware of their ALX, while at the same time learning how to tolerate and manage the ensuing uncertainty. This conclusion could be tested in future studies by ensuring that ALX and IU are regularly included as process of change variables in intervention trials that target anxiety.

While the results of the current study are clearly encouraging, it is important to acknowledge some important limitations. First, our sample size is modest and the group of adults is not representative of the wider adult autism community in terms of intellectual ability and core clinical difficulties. In this context, it is important that the 15 participants who dropped out after returning initial baseline data had lower verbal IQs and more significant social-communication difficulties than the participants who were retained in the study. This suggests that the online tools we examined may be useful only for autistic adults who do not have significant language or intellectual impairments. While there is clearly a need to further develop online tools to be more widely accessible to the autism community, it is also worth noting that higher IQ and cognitive ability have been associated with greater levels of anxiety in autism (see Vasa & Mazurek, 2015), so the fact that existing tools may help primarily cognitively able autistic adults still has important practical implications.

Another caveat is that groups were not matched on baseline levels of anxiety, and participants were largely self-selected in response to advertisement of the study. The baseline differences make the relative improvements in the different groups difficult to compare. Treatment benefits may have been overestimated in the CBT group where baseline levels of anxiety were most pronounced, whereas benefits in the MBT group may have been underestimated because baseline levels of anxiety in this group were generally lower. Some of these limitations could be addressed by examining the reliability and clinical significance of change at an individual level but future studies should nevertheless better control for baseline levels of anxiety. In terms of the fact that participants were self-selected in the current study, this may generally over-estimate treatment benefits because participants may have volunteered who have favourable opinions of CBT and MBT or who anticipate benefitting from taking part in the study and therefore report desired improvements in symptoms. Such biases may help explain why our WL group also demonstrated some reliable reductions in anxiety and improvements in broader clinical outcomes.

It is also important to acknowledge that our attempts to monitor treatment fidelity and engagement with the online tools were not entirely successful. As noted briefly in the methods section, we had provided participants in the active conditions with diaries to record how frequently they utilised the different strategies they learned throughout the active period and to note any thoughts or feedback they might have about the online tools. Unfortunately, many did not return these diaries, often because they were misplaced. Because the *Be Mindful* platform logs progress and because we arranged phone calls with participants on a weekly basis to ensure they were progressing through the programmes, we are confident that they did complete the programmes as intended. However, future studies would benefit from alternative formats of collecting more formal treatment fidelity information. It is likely more effective to integrate such data collection more directly with relevant online platforms, or to supplement such platforms with brief periodic electronic surveys regarding strategy utilisation and broader feedback.

Finally, given the pilot nature of this study, we elected to not pre-register the trial. This is an important next step in evaluating the efficacy of CBT and mindfulness with a larger sample.

In conclusion, it will be important to replicate and extend the current findings in larger-scale trials that overcome some of the current methodological limitations. It will also be important to further develop online mental health services that cater more specifically to the needs of autistic individuals. In face-to-face settings, concrete recommendations already exist for how therapies should be adapted for autistic individuals, for example, by incorporating special interests in sessions, ensuring that abstract concepts and metaphors are understood, and by providing extended psychoeducation about the nature of thoughts and emotions (e.g. Attwood, 2004; Kerns et al., 2016). Many of these adaptations should be feasible also for online support tools, and additional consideration may need to be given to how material is laid out and presented (e.g. audio-visual material vs written instructions, etc.). Such work is fortunately already underway, and the first autism-specific mobile app for managing anxiety was recently launched with critical input from autistic users (see https://www.autistica.org.uk/get-involved/molehill-mountain-app). This is a next step in translating the wealth of evidence that now exists about anxiety in autism, into mental health services and tools that are both effective and accessible.

## Supplemental Material

Gaigg_et_al._Autism_and_Mental_Health_SI_supplemental_material_Clean_Revisions – Supplemental material for Self-guided mindfulness and cognitive behavioural practices reduce anxiety in autistic adults: A pilot 8-month waitlist-controlled trial of widely available online tools Click here for additional data file. Supplemental material, Gaigg_et_al._Autism_and_Mental_Health_SI_supplemental_material_Clean_Revisions for Self-guided mindfulness and cognitive behavioural practices reduce anxiety in autistic adults: A pilot 8-month waitlist-controlled trial of widely available online tools by Sebastian B Gaigg, Paul E Flaxman, Gracie McLaven, Ritika Shah, Dermot M Bowler, Brenda Meyer, Amanda Roestorf, Corinna Haenschel, Jacqui Rodgers and Mikle South in Autism

## Notes

1. Although Nicholson et al. were not directly concerned with the association between alexithymia and anxiety in this article, they report in Supplementary Material a moderate (r = 0.4) correlation between anxiety and alexithymia in a group of 21 autistic adults.

2. Although our analyses focused on sub-scales of the Bermond-Vorst Alexithymia Questionnaire (BVAQ) and Five Facet Mindfulness Questionnaire (FFMQ), both of these questionnaires were administered in full to preserve the integrity of the measures.

3. Diagnostic (Autism-Spectrum Quotient (AQ), Social Responsiveness Scale (SRS), Autism Diagnostic Observation Schedule, Second Edition (ADOS-2)) and clinical (Wechsler Adult Intelligence Scale (WAIS)) data were not sought at baseline, partly because we felt that completing additional questionnaires would be less of a burden for participants once they were familiar with the general research process, and partly because it would have been difficult to arrange face-to-face appointments for ADOS and WAIS assessments without compromising our intended schedule for participant enrolment.

4. Reliable change (RC) can be calculated on the basis of the standard error and the internal reliability (i.e. Cronbach’s alpha) of the instrument at baseline. When measurements are taken across two time-points, the following formula quantifies the expected error of the difference (SE_diff_) of the scores (see Evans et al., 1998): SE_diff_ = SD*√2*√1 − r, where r is the reliability of the measure (Cronbach’s alpha). When the change between time-points exceeds 1.96 times, SE_diff_ RC can be inferred because it is unlikely that such change occurs by chance.
