# Supplementary material for: Self-guided mindfulness and cognitive behavioural practices reduce anxiety in autistic adults: A pilot 8-month waitlist-controlled trial of widely available online tools
Source: Autism. 2020 Apr 8;24(4):867–83. doi: 10.1177/1362361320909184 (PMC7418273; doi:10.1177/1362361320909184)
Supplement: Gaigg_et_al._Autism_and_Mental_Health_SI_supplemental_material_Clean_Revisions – Supplemental material for Self-guided mindfulness and cognitive behavioural practices reduce anxiety in autistic adults: A pilot 8-month waitlist-controlled trial of widely available online tools [file Gaigg_et_al._Autism_and_Mental_Health_SI_supplemental_material_Clean_Revisions.pdf]

Supplemental Material 1 (S1): ***Details of the 9 self-report questionnaire measures, including a Table summarising their Cronbach alpha internal consistencies***

1) The *General Anxiety Disorder-7* (GAD-7; Spitzer et al., 2006), is a seven-item questionnaire that asks participants to rate how bothered they feel by situations that are associated with feelings of anxiety and worry on a 4-point Likert scale. Examples of items include difficulties relaxing, becoming easily annoyed and fearing that something bad might happen at night. All items are rated from 0 (not all all) to 3 (nearly every day). Scores of 5, 10, and 15 are taken as the cut-off points for mild, moderate and severe anxiety, respectively.

2) The *Liebowitz Social Anxiety Scale* (LSAS; Fresco et al., 1987), is a 24-item questionnaire that measures the extent to which social phobia effects individuals. Each question is rated on two major sub scales – a fear/anxiety scale and an avoidance scale. Thus participants are asked to rate to what extent they feel fear or anxiety about certain situations and separately they indicate to what extent they try to avoid these situations. Examples include statements about participating in small groups, going out to a party, taking a test and other similar situations. Each item is rated from 0 (none) to 3 (Severe), with higher scores indicating higher social anxiety. A maximum score of 144 points can be obtained, with scores above 60 indicative of social anxiety disorder.

3) The *Beck's Anxiety Inventory* (BAI; Beck et al., 1988) is a 21-item measure, designed to capture primarily the physiological and behavioural symptoms of anxiety. Thus the questionnaire lists symptoms such as feeling numb or dizzy or feeling ones heart racing and participants are asked to indicate how much they are bothered by such symptoms. All items are rated from 0 (not at all) to 3 (severely) with higher scores indicating higher levels of anxiety. Scores over 15 are thought to indicate significant levels of anxiety.

4) The *State–Trait Anxiety Inventory – Trait version* (STAI-T; Spielberger, et al, 1983) is a 20-item measure of how a person feels on a daily basis (rather than over a specified recent period). Examples of items include “I am happy,” and “I feel inadequate” and all items are rated on a scale of 1 (almost never) to 4 (almost always) with higher scores indicating higher levels of anxiety.

5) The *Bermond-Vorst Alexithymia Questionnaire* (BVAQ-3; Vorst & Bermond, 2001) is a 20-item measure of alexithymia that comprises five subscales: Verbalizing, Fantasising, Identifying, Emotionalising and Analysing. The scale asks participants to rate how much certain statements apply to them and for the purposes of the current study, the Verbalize and Identify subscales were of particular interest, which includes statements such as “people often say I should talk about my feelings”; “I can express my feelings verbally”; “When I feel lousy, I know whether I am afraid or dejected or sad”. Ratings range from 1 (strongly disagree) to 5 (strongly agree) for each item and higher scores indicate a greater degree of difficulty identifying and describing own emotions.

6) The *Five Facet Mindfulness Questionnaire* (FFMQ; Baer, Smith, Hopkins, Krietemeyer, & Toney, 2006) is designed to measure the distinct features of mindfulness and has 39 items. The scale comprises five subscales: Observing, Describing, Acting with awareness, Non-judgmental attitudes toward inner experience, and Non-reactivity to inner experience. This latter sub-scale was of particular interest in the current study, which includes statements such as “I perceive my feelings and emotions without having to react to them”. Participants are asked to rate their agreement with the items on a 1 (never or rarely true) to 5 (very often or always true) scale with higher scores indicating higher levels of mindfulness.

7) The *Intolerance of Uncertainty Scale* (IUS-12; Carleton, Norton, & Asmundson, 2007) is a short 12-item scale that measures the extent to which participants avoid uncertainties and/or experience uncertainties as distressing. Statements participants are asked to rate include "unforeseen events upset me greatly" and "when I am uncertain I can't act very well". Items are rated on a scale of 1 (not at all characteristic of me) to 5 (entirely characteristic of me) with higher scores indicating greater intolerance of uncertainty.

8) The *The Hospital Anxiety and Depression Scale* (HADS; Zigmond & Snaith, 1983) is used to determine levels of anxiety and depression. In the current study, the depression subscale was of interest, which comprises 7 statements that participants are asked to rate on a 0 (not at all / least frequent) to 3 (definitely / most frequent) scale. Examples of the statements include "I am able to laugh and see the funny side of things", "I still enjoy the things I used to enjoy".

9) Finally, the *Clinical Outcomes in Routine Evaluation–Outcome Measure* (CORE-OM; Evans et al., 2000) is used to measure overall psychological distress and clinical functioning across four domains: Wellbeing (e.g., "I have felt OK about myself"; "I have felt like crying"), Functioning (e.g., "I have felt able to cope when things go wrong"; "I have been able to do most things I need to"), Symptoms (e.g., "I have felt tense, anxious or nervous"; "I have been troubled by aches, pains or other physical problems") and Risk (e.g., "I have been physically violent to others"; "I made plans to end my life"). Participants are asked to rate in how far each item is true for them on scale of 0 (Not at all) to 4 (Most of the time) with higher scores indicating greater levels of concern.

Cronbach's Alpha for all self-report questionnaires. The measures are listed in the order in which they were administered.

Questionnaire	*Cronbach's alpha*
Beck's Anxiety Inventory (BAI)	0.92
Liebowitz Social Anxiety Scale (LSAS)	0.97
Hospital Anxiety and Depression Scale (HADS-D)[a]	0.86
Clinical Outcomes in Routine Evaluation Outcome Measure (CORE-OM)	0.95
General Anxiety Disorder - 7 (GAD)	0.93
State-Trait Anxiety Inventory - Trait (STAI-T)	0.94
Intolerance of Uncertainty Scale-12 (IUS-12)	0.92
Five-Facet Mindfulness Questionnaire (FFMQ-NR)	0.83
Bermond Vorst Alexithymia Questionnaire (BVAQ-ID)[a]	0.73

Note: For the HADS, BVAQ and FFMQ the full scales were administered but Cronbach's alpha is reported here only for the relevant sub-scales that were the focus of the analyses. The respective values for the full scales were 0.92 for HADS, 0.77 for the BVAQ and 0.86 for the FFMQ.

Supplemental Material 2 (S2): *Details of the online MBT (Be Mindful) and CBT (Serenity) programmes.*

The online MBT course *Be Mindful* (https://www.bemindfulonline.com/) comprises four modules that present a total of 10 different exercises. Briefly, modules 1 and 2 teach participants how to bring their awareness to the present moment to notice sensations in their bodies and their environment. Participants are encouraged to practice this mindful attention whilst sitting or lying still as well as during routine daily activities such as eating or walking. Modules 3 and 4 then build on these practices to encourage participants to be mindful also about their thoughts and feelings, simply becoming aware of them with curiosity and to accept them without judgement or trying to change them. The four modules are set up to be completed over 4 weeks with automated e-mail reminders sent at regular intervals to invite participants to access new materials and exercises. Participants could, however, move through the exercises at their own pace and for the purposes of the current study we asked

participants to try to progress through the course in 6-8 weeks. At the end of the course, they gained access to a printed guide designed to help practice mindfulness every day.

The online CBT course *Serenity* (https://serene.me.uk) is structured into 8 sections that each provide information and exercises through a series of 12 illustrated slides that teach people strategies on how to identify and manage feelings of anxiety. Briefly, section 1 introduces participants to the nature of anxiety and explains the biological processes involved, including an overview of the fight, flight and freeze response. Section 2 guides participants through ways in which they can change the way they think and respond to situations, and an exercise encourages them to think about how things would change if their anxiety levels were lower. The third section then introduces the principles of CBT by illustrating that our thoughts, feelings and behaviours are connected and that changing one (e.g., thoughts) can influence the others (feelings & behaviour). Sections 4 – 6 then teach individuals strategies for managing anxious feelings and stress through a combination of relaxation, grounding and re-appraisal techniques. Finally, section 7 provides problem-solving guidance as a way of managing common situational triggers of stress and anxiety, and section 8 ends with some broader tips and techniques to stay well. Unlike the *Be Mindful* course, *Serenity* does not require participants to create an account or log in, and there are no automated reminders to progress participants through the different sections and exercises.

Supplemental Material 3 (S3): *Summary of the results of the regression analyses of the baseline data, which examined the contributions of Intolerance of Uncertainty (IU) and* non-reactive thinking (NR) *to the different measures of anxiety.*

Dependent	Model (adjusted R^2; F test)	Predictors	Beta	t	*p*
GAD-7	R^2 = .39; F(2,51) = 17.80, p <.001	IU	.412	3.37	.001
		FFMQ-NR	-.333	2.73	.009
LSAS-Fear	R^2 = .33; F(2,51) = 13.97, p <.001	IU	.548	4.28	<.001
		FFMQ-NR	-.089	0.69	.492
LSAS-Avoid	R^2 = .23; F(2,51) = 8.69, p =.001	IU	.515	3.74	<.001
		FFMQ-NR	.023	.168	.867
STAI-T	R^2 = .61; F(2,51) = 41.84, p <.001	IU	.362	3.70	.001
		FFMQ-NR	-.549	5.61	<.001
BAI	R^2 = .35; F(2,51) = 15.31, p <.001	IU	.433	3.45	.001
		FFMQ-NR	-.273	2.17	.034

Supplemental Material 4 (S4): *The Percentage of participants demonstrating reliable change (RC) and clinically significant change (CSC) on each of the four anxiety measures on which clinical caseness was demonstrated at baseline.*

		GAD-7				LSAS				STAI-T				BAI			
		n	T1-T2	T1-T3	T1-T4	*n*	T1-T2	T1-T3	T1-T4	*n*	T1-T2	T1-T3	T1-T4	*n*	T1-T2	T1-T3	T1-T4
RCI	MBCT	7	14.3%	42.9%	28.6%	10	30.0%	50.0%	30.0%	11	18.2%	18.2%	36.4%	9	0.0%	33.0%	22.0%
	CBT	6	66.7%	66.7%	33.3%	9	22.2%	55.6%	33.3%	8	12.5%	25.0%	25.0%	8	25.0%	50.0%	15.0%
	WL	12	16.7%	25.0%	16.7%	14	21.4%	21.4%	35.7%	14	7.1%	28.6%	14.3%	13	0.0%	15.4%	23.0%
CSC	MBCT	4	25.0%	50.0%	0.0%	6	16.7%	33.0%	0.0%	7	14.3%	28.6%	28.6%	4	0.0%	50.0%	25.0%
	CBT	5	40.0%	40.0%	40.0%	4	0.0%	25.0%	25.0%	5	20.0%	20.0%	40.0%	6	33.3%	16.7%	16.7%
	WL	8	25.0%	12.5%	0.0%	9	11.1%	11.1%	22.2%	11	9.1%	27.3%	18.2%	9	0.0%	11.1%	22.2%
